# Chirality and Relativistic Effects in Os_3_(CO)_12_

**DOI:** 10.3390/molecules26113333

**Published:** 2021-06-01

**Authors:** Maxim R. Ryzhikov, Irina V. Mirzaeva, Svetlana G. Kozlova, Yuri V. Mironov

**Affiliations:** Nikolaev Institute of Inorganic Chemistry SB RAS, 3, Acad. Lavrentiev Ave., 630090 Novosibirsk, Russia; maximato@mail.ru (M.R.R.); dairdre@gmail.com (I.V.M.); sgk@niic.nsc.ru (S.G.K.)

**Keywords:** structure of the Os_3_(CO)_12_ clusters, chirality, relativistic effects, parity violating energy difference, quantum chemistry

## Abstract

The energy and structural parameters were obtained for all forms of the carbonyl complex of osmium Os_3_(CO)_12_ with D_3h_ and D_3_ symmetries using density functional theory (DFT) methods. The calculations took into account various levels of relativistic effects, including those associated with nonconservation of spatial parity. It was shown that the ground state of Os_3_(CO)_12_ corresponds to the D_3_ symmetry and thus may be characterized either as left-twisted (D_3S_) or right-twisted (D_3R_). The D_3S_↔D_3R_ transitions occur through the D_3h_ transition state with an activation barrier of ~10^–14^ kJ/mol. Parity violation energy difference (PVED) between D_3S_ and D_3R_ states equals to ~5 × 10^−10^ kJ/mol. An unusual three-center exchange interaction was found inside the {Os_3_} fragment. It was found that the cooperative effects of the mutual influence of osmium atoms suppress the chirality of the electron system in the cluster.

## 1. Introduction

It is well known that enantiomerism is directly related to the origin of life on Earth. Many essential biological and chemical processes are stereoselective, involving only one enantiomer, which exists independently from its chiral counterpart. Separation of one enantiomer from another is not only important for practical use, but also sparks much interest as a fundamental problem, which constantly attracts research attention to the spatial and electronic structure of enantiomers [1,2,3]. In particular, especially interesting is how the parity violating weak nuclear forces may manifest in chiral molecules and may be somehow responsible for the choice of which enantiomer would prevail in living organisms [4,5,6,7].

Molecules with D_3_ symmetry are particularly interesting because they are characterized either with left-handed (D_3S_) or with right-handed (D_3R_) torsion, which allows these structures to be treated as chiral enantiomers. Previously, the detailed analysis of the 1,4-diazabicyclo[2.2.2]octane (DABCO) molecule with D_3S_ (left-twisted), D_3R_ (right-twisted), and D_3h_ (untwisted) symmetries in gas phase and in Metal-organic Frameworks (MOFs) was performed [8,9]. There were expectations that MOFs with a DABCO linker may undergo a number of phase transitions related to enantiomers ordering [2,10], and that cooperative effects in such systems may help the experimental search of molecular parity violation effects [7,9,11]. However, the energy barrier between the D_3S_ and D_3R_ states of DABCO appears to be very low (<100 J/mol in gas phase [8] and ~5 kJ/mol in MOF [9]), which makes MOFs with DABCO difficult for experimental studies of such effects.

**Figure 1:**
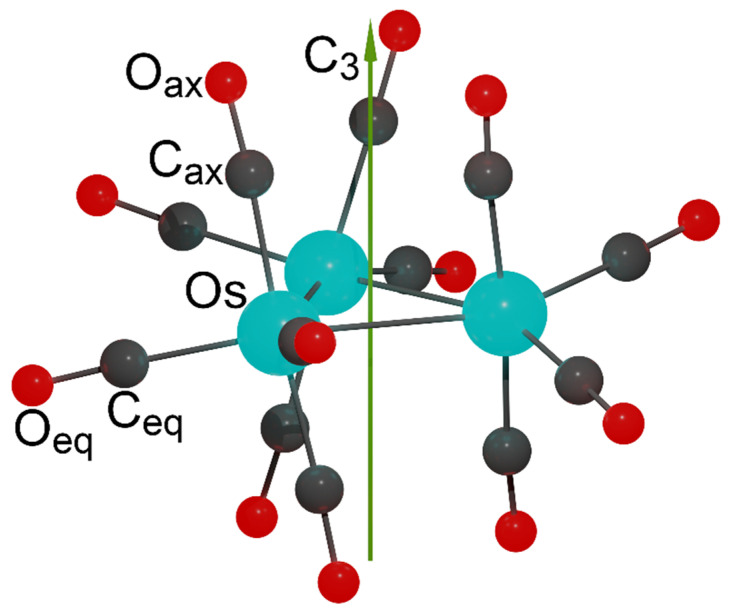
The structure of Os_3_(CO)_12_ cluster corresponding to the local minimum on PES.

Similarly to the DABCO molecule, trinuclear transition metal cluster complexes may also have D_3_ symmetry. A well-known carbonyl osmium complex Os_3_(CO)_12_ (Figure 1) is widely used in synthesis in organometallic and inorganic chemistry [12,13,14]. It acts as a catalyst for a wide range of reactions [15]. It was considered as a model system for studies of mechanisms of photoinduced reactions [16,17,18] and vibrational and NMR spectroscopy [19,20,21]. In crystal, which is reported to have a monoclinic space group P2_1_/n, the Os_3_(CO)_12_ molecules show approximate (pseudo-) D_3h_ symmetry, where the three Os atoms form an almost equilateral triangle [22]. Previous detailed quantum chemical studies of structure and interatomic interactions in the Os_3_(CO)_12_ cluster have shown that in the gas phase, the ground state has the D_3_ symmetry, while the D_3h_ symmetry refers to a transition state. It was also found that there is another transition state with C_2v_ symmetry and higher energy, which, however, may occur at high temperatures [23,24,25].

In the present study, for the first time, the Os_3_(CO)_12_ cluster was considered as a chiral compound. We investigated what exactly is responsible for the stabilization of the twisted D_3_ structure compared to the untwisted D_3h_ one. We also evaluated the impact of relativistic effects on the structure and energetics of the Os_3_(CO)_12_ clusters, including the parity violation energy shift due to the weak nuclear forces. We expected that due to the cooperative effects, such trinuclear transition metal clusters may become a good family of systems for the experimental molecular parity violation search.

## 2. Results and Discussion

### 2.1. Structure and Energetics of Os_3_(CO)_12_

**Figure 2:**
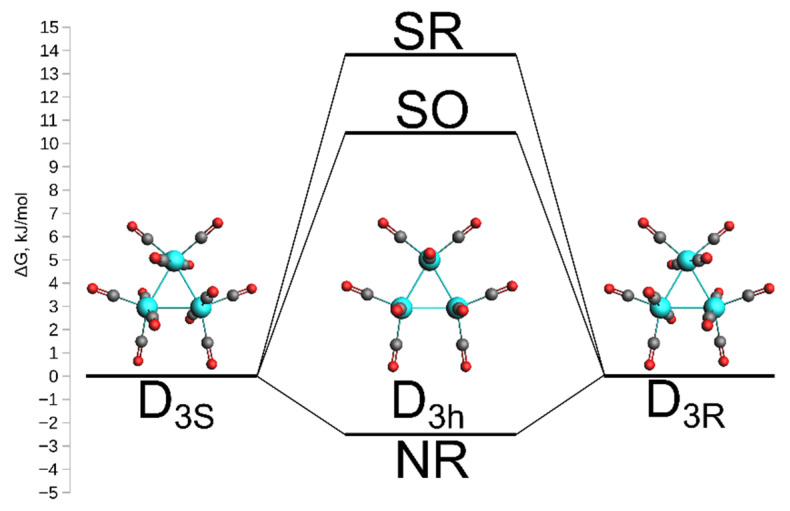
The barrier height for the D_3S_↔D_3R_ enatiomerization through the D_3h_ transition state calculated at nonrelativistic (NR), scalar (SR) and spin-orbit (SO) relativistic levels of theory.

The analysis of the interatomic distances in Os_3_(CO)_12_ (Table 1 and Table S1) shows that the best agreement between calculated and experimental X-ray diffraction (XRD) structures [22,26] was observed for the calculations with relativistic effects. Moreover, the difference between scalar (SR) and spin–orbit (SO) relativistic methods is rather small. However, they both are very different from the nonrelativistic (NR) level of theory. In this case, the D_3h_ is the local minimum without imaginary frequencies in vibrational spectrum, while the D_3_ symmetry refers to just some point on potential energy surface (PES) and is characterized with two imaginary frequencies. The energy difference between the structures with D_3_ and D_3h_ symmetries calculated at the relativistic level (SR and SO) indicates that the D_3_ state is the local minimum on the potential energy surface. At both SR and SO levels of theory, there are no imaginary frequencies for the structure with D_3_ symmetry, while D_3h_ is a transition state with a single imaginary frequency. Gibbs free energy differences between D_3_ and D_3h_ structures are 13.8 kJ/mol and 10.5 kJ/mol for SR and SO levels of theory, respectively (Figure 2). Previous calculations with relativistic effective core potential (ECP) also indicated that the D_3_ state is a local minimum [25]. Thus, it can be concluded that the D_3_ is the local minimum in the gas phase and the taking into account the relativistic effects at least at ECP level is necessary for correct description of the system.

The main difference between experimental XRD data and relativistic calculations (for both SR and SO methods) is found in the dihedral angles ∠C-Os-Os-C и ∠C_3axes_-Os-C, which characterize the degree of the twisting of the Os_3_(CO)_12_ structure. Such a difference could be related to the packing effects in the solid state. In order to compare the packing energy with the barrier between the cluster structures with D_3S_ and D_3R_ symmetries, the periodic calculations were performed. The packing energy is ~ 44 kJ/mol per cluster, while the barrier is ~10 kJ/mol. Thus, the packing effect is big enough to suppress the twisting of the Os_3_(CO)_12_ clusters in the solid state.

The energy difference between D_3_ and D_3h_ structures characterizes the barrier between D_3S_ and D_3R_ enantiomers of Os_3_(CO)_12_ cluster. Without consideration of parity violating interactions, D_3S_ and D_3R_ enantiomers have exactly the same energy and electronic structure. The geometries of D_3S_ and D_3R_ enantiomers differ only by the sign of the Z coordinates of the atoms (Table S1). Thus, any of the enantiomers can be used to analyze the interactions in the cluster.

The barrier between D_3S_ and D_3R_ is low (~10–14 kJ/mol). Thus, similarly to the DABCO molecule [1], at room temperature there should be a dynamical equilibrium between D_3S_ and D_3R_ structures resulting in a quasi-D_3h_ structure.

### 2.2. Characterization of the Interactions in the Os_3_(CO)_12_ Cluster

The energy decomposition analysis (EDA) of the interactions between Os(CO)_4_ fragments in the Os_3_(CO)_12_ cluster show that the orbital interactions’ contributions to stabilization of the D_3_ structure are larger than for the D_3h_ form (Table 2). On the contrary, the steric interactions destabilize D_3_ compared to the D_3h_. The distortions of the Os(CO)_4_ fragments in the clusters, as well as dispersion interactions, have only minor impacts on the energy difference between structures with D_3_ and D_3h_ symmetries and, in general, compensate each other. Thus, the stronger orbital interactions between the Os(CO)_4_ fragments are the main factors that are responsible for the stability of the D_3_ form.

**Figure 3:**
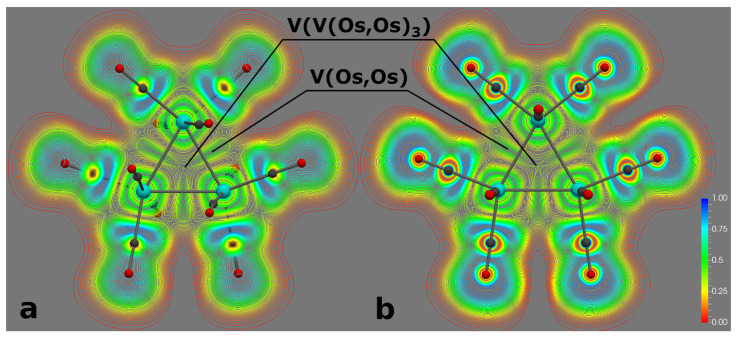
ELF slice planes for Os_3_(CO)_12_ clusters with D_3_ (**a**) and D_3h_ (**b**) symmetries.

**Figure 4:**
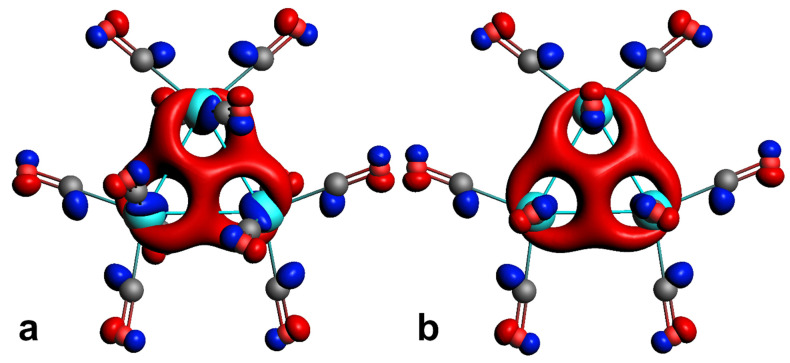
HOMO-2 orbitals for Os_3_(CO)_12_ clusters with D_3_ (**a**) and D_3h_ (**b**) symmetries.

ELF analysis of the interactions in the osmium triangle showed the presence of the three valence V(Os, Os) basins (Figure 3), which indicate the covalency of the interactions between Os atoms in both D_3_ and D_3h_ structures of Os_3_(CO)_12_ cluster. Also, both structures are characterized with the single trisynaptic basin in the center of Os_3_ triangle. However, this basin does not have common borders with any of the core basins (Figures S1 and S2). It shares borders only with valence V(Os, Os) basins and, thus, this basin indicates the electron exchange between V(Os, Os) basins. To our best knowledge, it is the first example of such ELF topology. Formally, this basin should be classified as V(V(Os, Os)_3_). In the list of the molecular orbitals, the HOMO-2 (Figure 4) could be the one responsible for this V(V(Os, Os)_3_) basin. Note that the populations of the V(V(Os, Os)_3_) basins are very small (0.03e and 0.01e for D_3_ and D_3h_ structures, respectively), however it should provide the exchange between V(Os, Os). This assumption is consistent with results of previous QTAIM analysis about three center interactions in Os_3_(CO)_12_ cluster [23].

### 2.3. Chirality and Parity Violation in Os_3_(CO)_12_

Our calculations indicate that relativistic effects define the D_3_ symmetry of the ground state of Os_3_(CO)_12_ molecule, which has the two variants: left-twisted D_3S_ and right-twisted D_3R_. The next level of relativistic effects would be the calculation of the contributions of the nuclear weak forces to the energy of the system. These forces violate spatial parity symmetry; thus, they make the energy of enantiomers slightly different. Experimental evidence of such effect have been observed on nuclear and atomic levels [27]. However, parity violation effects on molecular level have not been detected experimentally yet [7]. Thus, it is important to look for good candidates for experimental search of molecular parity violation.

The major contribution to PVED comes from the nuclear-spin-independent part of the parity-violating Hamiltonian [28]:

(1) $$H_{PV}^{SI}=\frac{G_{F}}{2\sqrt{2}}\sum_{n}^{N_{nuc}}\left(Q_{W}\left(n\right)\gamma^{5}\rho_{n}\left(r\right)\right),$$

where *G_F_* is the Fermi-coupling constant with a value of 2.22255 × 10^−14^ a.u.; *γ^5^* is the fifth Dirac gamma matrix, which refers to the electron chirality operator; *ρ_n_*(*r*) is the normalized nucleon density; *N_nuc_* is the number of nuclei in the molecule; and *Q_W_*(*n*) is the weak nuclear charge of nucleus *n*, which depends on the number of protons *Z*(*n*) and neutrons *N*(*n*) in the nucleus. It is given by the expression:

(2) $$Q_{W}\left(n\right)=Z\left(n\right)\left(1-4\sin^{2}\theta_{W}\right)-N\left(n\right)$$

with *θ_W_* being the Weinberg angle.

Previous Density Functional Theory (DFT), Hartree-Fock method (HF), and Many Body Perturbation Theory (MBPT) calculations for compounds of various six-row elements showed rather low values of PVED: ~3 × 10^−12^ kJ/mol for Hg [9], ~6 × 10^−11^ kJ/mol for Re and ~2 × 10^−10^ for Os [29], up to ~2 × 10^−10^ for Bi [30], and the largest would be ~8 × 10^−9^ kJ/mol for the hypothetical analogue of hydrogen peroxide—Po_2_H_2_ [31]. However, some of those estimations were made using approximate two-component relativistic methods, while our previous work showed that results of two-component PVED calculations in some systems may differ from the four-component results by up to 30% [11]. Therefore, in this work we used only fully relativistic four-component methods for calculations of PVED.

We may try to estimate the characteristic PVED values for Os compounds. As weak interactions are local and largely depend on the atomic number, the main contribution would be from the heaviest nucleus in the molecule (in our case Os) and may be estimated as follows [32]:

(3) $$\mathrm{PVED}\;\approx \;G_{F}\alpha^{3}Z^{4}NK_{r}\eta$$

where *α* is the fine-structure constant, *Z* is the charge of the heaviest nucleus in the molecule, *N* is the number of neutrons in the heaviest nucleus in the molecule, *K_r_* is some enhancement factor for relativistic effects (for the first row atoms *K_r_* ~ 1, while for the heavier period six elements *K_r_* ~10), and *η* is the dimensionless molecular asymmetry factor. The estimations of typical *η* in organic molecules fall in the range from ~10^−9^ [33] to ~10^−2^ [34]. Therefore, according to Equation (3), PVED in Os compounds may be preliminarily estimated as ~8 × 10^−16^–~8 × 10^−9^ kJ/mol.

**Figure 5:**
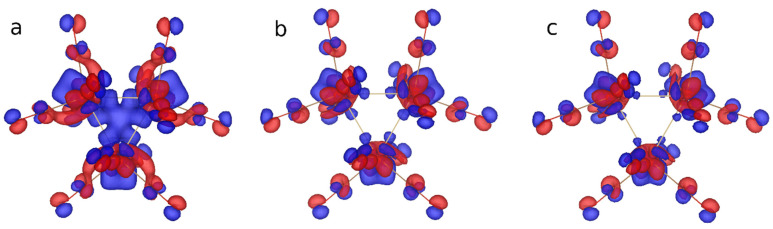
Electron chirality density (*γ^5^*) calculated at HF (**a**), DFT PBE0 (**b**), and PBE (**c**) levels of theory.

Especially interesting are the possible cooperative effects in trinuclear Os_3_(CO)_12_ clusters. The contributions from the three Os atoms may just sum up, but also it may either additionally increase or suppress the chirality of the system. To extract such effects, we may compare the PVED values for Os_3_(CO)_12_ with some mononuclear reference system. We chose the hypothetical OsOSSeTe complex, which is analogous to the osmium tetraoxide. The results are shown in Table 3. The PVED values for Os_3_(CO)_12_ are closer to the upper estimate for Os compounds. We can see that Generalized Gradient Approximation (GGA) methods consistently give lesser values than HF, while hybrid and range-separated methods predictably give half-way values. This is supported by the electron chirality density pictures (Figure 5). The most surprising is the strong dependence of PVED for OsOSSeTe from the method used. This is due to the fact that contribution from the second heavy atom Te is of the opposite sign and DFT GGA methods, for some reason, redistribute the electron chirality in such a way that it becomes much higher on the Te atom. However, even the largest PVED value for OsOSSeTe (HF) is an order of magnitude less than the corresponding value for Os_3_(CO)_12_.

**Figure 6:**
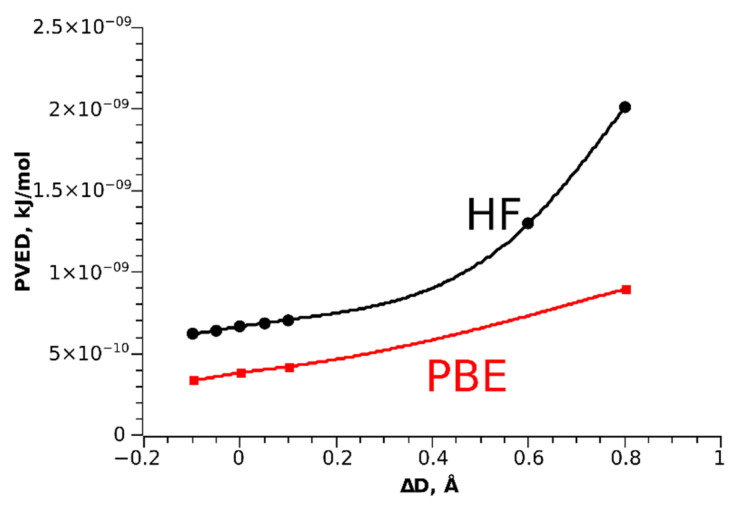
PVED as a function of Os-Os distance change, zero refers to the equilibrium Os-Os distance.

Another model for testing the cooperative effects is to calculate PVED for stretched and contracted versions of the Os_3_(CO)_12_ cluster, where only the Os-Os distances change. We may expect the decrease of geometric chirality parameter *η* with the increase of the Os-Os distances. However, the calculated PVED values actually grow with increase of the Os-Os distances (Figure 6) for both HF and DFT PBE methods. This indicates that stronger interaction between Os atoms rather suppresses the electron chirality in the system.

## 3. Materials and Methods

### Computational Details

Geometry optimization of the Os_3_(CO)_12_ cluster with D_3_ and D_3h_ symmetries was performed in ADF2020 program [40,41] with all-electron Slater’s type TZ2P basis set [42], TPSSh [43] density functional, and Grimme D4(EEQ) [44] dispersion corrections. The calculations were performed in the gas phase. In order to analyze the influence of the relativistic effects on the geometry of the cluster, the calculations were performed without relativistic approximation (NR), with scalar relativistic (SR), and with spin-orbit (SO) zero-order regular approximation (ZORA) [45,46]. To minimize the basis set superposition error (BSSE), the single point calculations at TPSSh + D4(EEQ) + SR/QZ4P level of theory were performed for the energy decomposition analysis (EDA) [47] and electron localization function calculations (ELF) [48,49]. ELF analysis was performed in the dgrid-4.6 program [50] on the discreet mesh with the step of 0.05 a.u.

The periodic calculation of the Os_3_(CO)_12_ cluster in the solid state were performed in the BAND2020 program [51,52] with SCAN density functional [53], all-electron TZP basis set, and SR ZORA approximation. The starting geometry for the periodic calculations was taken from the experimental XRD structural data [22].

Calculations of parity-violating energy difference (PVED) between left- and right-twisted structures of Os_3_(CO)_12_ in the gas phase were performed in Dirac-19 [32,54] with a fully relativistic four-component Dirac–Coulomb Hamiltonian. An all-electron double-zeta dyall.ae2z relativistic basis set [55], in combination with Hartree–Fock and various DFT methods, was used. To highlight the cooperative effect of three Os atoms, we compared the PVED for Os_3_(CO)_12_ with PVED for a hypothetical compound OsOSSeTe, where there was only one Os atom. The structure of OsOSSeTe in the gas phase was also optimized in ADF2020 at the TPSSh + D4(EEQ) + SR/QZ4P level of theory.

## 4. Conclusions

In our study, for the first time, the chirality of the Os_3_(CO)_12_ was investigated. With an account of relativistic effects, the twisted structure with the D_3_ symmetry refers to the local minimum on potential energy surface, while the nonchiral D_3h_ structure refers to the transition state with a single imaginary frequency. The chiral D_3_ structure is, apparently, stabilized by the relativistic effects. Moreover, the EDA analysis of the interactions in Os_3_(CO)_12_ showed that D_3_ structure has the stronger orbital interactions between Os(CO)_4_ fragments.

The ELF topological analysis showed the unusual pattern of the basins. The trisynaptic basin in the center of Os triangle has no borders with any of the core basins, apparently, indicating the exchange between disynaptic V(Os, Os) basins.

We also estimated the PVED values for the Os_3_(CO)_12_ cluster. At present, the obtained values are the largest predicted, with the exception of the hypothetical H_2_Po_2_ system [31]. Moreover, the study of cooperative effects in PVED of Os_3_(CO)_12_ clusters showed that the stronger interaction between Os atoms rather reduces the electron chirality in the system. Knowing that, we may hope to construct in future some molecular system with even larger PVED.

## Figures and Tables

**Table 1:** Interatomic distances (Å), dihedral angles (º), energy differences (kJ/mol), and number of imaginary frequencies (n*i*) for Os_3_(CO)_12_ clusters with D_3_ and D_3h_ symmetries. Error estimates shown in parentheses for average (<x>) distances or angles are the exterior estimates of the precision of the average value given by [Ʃ(<x> − x)^2^/(m^2^ − m)]^1/2^ (m is the number of x values) [22]. The TPSSh + D4(EEQ)/TZ2P and MPW1PW91/SDD are the theoretical levels used for geometry optimization (for details see Section 3.1). NR, SR, SO and ECP are the relativistic levels used in quantum chemical calculations. The average experimental distances and angles were taken from X-ray diffraction (XRD) data [22,26].

	TPSSh + D4(EEQ)/ TZ2P			TPSSh + D4(EEQ)/ TZ2P			XRD [22]	XRD [26]	MPW1PW91 /SDD [25]	
Symmetry	D_3_			D_3h_			Pseudo-D_3h_	Pseudo-D_3h_	D_3_	D_3h_
Relativity level	NR	SR	SO	NR	SR	SO			ECP	ECP
d(Os-Os)	2.808	2.869	2.869	2.801	2.883	2.884	<2.877>(3)	<2.881>(4)	2.895	2.907
d(Os-C_eq_)	1.968	1.916	1.914	1.966	1.915	1.914	<1.912>(7)	<1.919>(36)	1.917	1.917
d(Os-C_ax_)	2.001	2.11	1.955	1.992	1.954	1.953	<1.946>(6)	<1.973>(12)	1.953	1.95
∠C-Os-Os-C	37.2	29.3	29.2	0	0	0	<2.1>(5)	<1.3>(1.3)	31.4	0
∠C_3axes_-Os-C	21.2	16.8	16.8	0	0	0	<1.2>(5)	<0.7>(1.0)	18	0
ΔE	0	0	0	−3.0	7.5	7.7	-	-	−2.3	0
ΔG	0	0	0	−2.5	13.8	10.5	-	-	-	-
n*i*	2	0	0	0	1	1	-	-	0	1

**Table 2:** Results of energy decomposition analysis (EDA) for interactions between Os(CO)_4_ fragments in D_3_ and D_3h_ forms of Os_3_(CO)_12_ calculated at TPSSh + D4(EEQ) + SR/QZ4P//TPSSh + D4(EEQ) + SR/TZ2P level of theory. The formation energies of the fragments (E_frag_(Os(CO)_4_) as well as energies of steric (E_Steric_), orbital (E_Orbital_), dispersion (E_Disp_) and total (E_Int_) interactions between fragments are given in kJ/mol.

	E_frag_(Os(CO)_4_)	E_Steric_	E_Orbital_	E_Disp_	E_Int_
D_3_	−7838.3	520.1	−1191.6	−118.4	−789.9
D_3h_	−7839.6	489.5	−1153.1	−114.2	−778.2
∆(D_3_−D_3h_)	1.3	30.5	−38.5	−4.2	−11.7

**Table 3:** PVED (kJ/mol) calculated with different methods: Hartree-Fock (HF) and DFT with functionals PBE [35], PBE0 [36], BLYP [37], B3LYP [38], CAMB3LYP [39].

	HF	PBE	PBE0	BLYP	B3LYP	CAMB3LYP
Os_3_(CO)_12_	6.66 × 10^−10^	3.87 × 10^−10^	4.61 × 10^−10^	4.14 × 10^−10^	4.77 × 10^−10^	5.51 × 10^−10^
OsOSSeTe	4.08 × 10^−11^	5.11 × 10^−14^	2.23 × 10^−13^	3.40 × 10^−14^	1.17 × 10^−13^	1.35 × 10^−12^

## Data Availability

The data presented in this study are available in supplementary material.

## Supplementary Materials

The following are available online, Table S1: Coordinates of the Os_3_(CO)_12_ cluster optimized with Non Relativistic (NR), Scalar Relativistic (SR) and Spin-Orbit (SO) approximations at TPSSh + D4(EEQ)/TZ2P level of theory, Figure S1: ELF basins for Os_3_CO_12_ clusters with D_3_ (top) and D_3h_ (bottom) symmetries, Figure S2: Critical points between V(Os, Os) and V(V(Os, Os)_3_) basins.

## Sample Availability

Samples of the compounds are not available from the authors.
