# Supplementary material for: Chirality and Relativistic Effects in Os3(CO)12
Source: Molecules. 2021 Jun 1;26(11):3333. doi: 10.3390/molecules26113333 (PMC8199560; doi:10.3390/molecules26113333)
Supplement: Supplementary file 1 [file molecules-26-03333-s001.zip › molecules-1240919-SI.pdf]

Supporting Information to article:

Chirality and relativistic effects in $Os_3(CO)_{12}$

Ryzhikov M.R., Mirzaeva I.V., Kozlova S.G., Mironov Yu.V.

Nikolaev Institute of Inorganic Chemistry, Siberian Branch, Russian Academy of Sciences, Lavrentyev Av., 3, RU-630090 Novosibirsk, Russia

Table S1. Coordinates of the $Os_3(CO)_{12}$ cluster optimized with Non Relativistic (NR), Scalar Relativistic (SR) and Spin-Orbit (SO) approximations at TPSSh+D4(EEQ)/TZ2P level of theory.

D_{3R} NR

Os	1.621322	0.000000	0.000000
Os	-0.810661	1.404106	0.000000
Os	-0.810661	-1.404106	0.000000
O	3.688772	-2.214694	-0.702853
O	0.073595	4.301917	-0.702853
O	-3.762367	-2.087223	-0.702853
O	3.688772	2.214694	0.702853
O	-3.762367	2.087223	0.702853
O	0.073595	-4.301917	0.702853
O	1.527742	1.161842	-2.914438
O	-1.770056	0.742142	-2.914438
O	0.242314	-1.903985	-2.914438
O	1.527742	-1.161842	2.914438
O	0.242314	1.903985	2.914438
O	-1.770056	-0.742142	2.914438
C	2.920372	-1.414761	-0.428661
C	-0.234967	3.236497	-0.428661
C	-2.685405	-1.821736	-0.428661
C	2.920372	1.414761	0.428661
C	-2.685405	1.821736	0.428661
C	-0.234967	-3.236497	0.428661
C	1.469745	0.722848	-1.859265
C	-1.360877	0.911413	-1.859265
C	-0.108868	-1.634261	-1.859265
C	1.469745	-0.722848	1.859265
C	-0.108868	1.634261	1.859265
C	-1.360877	-0.911413	1.859265

D_{3R} SR

Os	1.656511	0.000000	0.000000
Os	-0.828255	1.434581	0.000000
Os	-0.828255	-1.434581	0.000000
O	3.586378	-2.280508	-0.672202
O	0.181788	4.246149	-0.672202

O	-3.768167	-1.965641	-0.672202
O	3.586378	2.280508	0.672202
O	-3.768167	1.965641	0.672202
O	0.181788	-4.246149	0.672202
O	1.608461	0.909453	-2.961923
O	-1.591840	0.938242	-2.961923
O	-0.016621	-1.847695	-2.961923
O	1.608461	-0.909453	2.961923
O	-0.016621	1.847695	2.961923
O	-1.591840	-0.938242	2.961923
C	2.863136	-1.435286	-0.394341
C	-0.188574	3.197191	-0.394341
C	-2.674562	-1.761906	-0.394341
C	2.863136	1.435286	0.394341
C	-2.674562	1.761906	0.394341
C	-0.188574	-3.197191	0.394341
C	1.568655	0.564249	-1.871147
C	-1.272981	1.076370	-1.871147
C	-0.295673	-1.640619	-1.871147
C	1.568655	-0.564249	1.871147
C	-0.295673	1.640619	1.871147
C	-1.272981	-1.076370	1.871147

D_{3R} SO

Os	1.656208	0.000000	0.000000
Os	-0.828104	1.434318	0.000000
Os	-0.828104	-1.434318	0.000000
O	3.586332	-2.278391	-0.671421
O	0.179978	4.245051	-0.671421
O	-3.766311	-1.966659	-0.671421
O	3.586332	2.278391	0.671421
O	-3.766311	1.966659	0.671421
O	0.179978	-4.245051	0.671421
O	1.609504	0.909033	-2.960993
O	-1.591998	0.939354	-2.960993
O	-0.017506	-1.848388	-2.960993
O	1.609504	-0.909033	2.960993
O	-0.017506	1.848388	2.960993
O	-1.591998	-0.939354	2.960993
C	2.862431	-1.433353	-0.394099
C	-0.189895	3.195615	-0.394099
C	-2.672536	-1.762262	-0.394099
C	2.862431	1.433353	0.394099
C	-2.672536	1.762262	0.394099
C	-0.189895	-3.195615	0.394099
C	1.569652	0.563535	-1.870245
C	-1.272862	1.077591	-1.870245
C	-0.296790	-1.641126	-1.870245
C	1.569652	-0.563535	1.870245
C	-0.296790	1.641126	1.870245
C	-1.272862	-1.077591	1.870245

D_{3S} NR

Os	1.621322	0.000000	0.000000
Os	-0.810661	1.404106	0.000000
Os	-0.810661	-1.404106	0.000000
O	3.688772	-2.214694	0.702853
O	0.073595	4.301917	0.702853
O	-3.762367	-2.087223	0.702853
O	3.688772	2.214694	-0.702853
O	-3.762367	2.087223	-0.702853
O	0.073595	-4.301917	-0.702853
O	1.527742	1.161842	2.914438
O	-1.770056	0.742142	2.914438
O	0.242314	-1.903985	2.914438
O	1.527742	-1.161842	-2.914438
O	0.242314	1.903985	-2.914438
O	-1.770056	-0.742142	-2.914438
C	2.920372	-1.414761	0.428661
C	-0.234967	3.236497	0.428661
C	-2.685405	-1.821736	0.428661
C	2.920372	1.414761	-0.428661
C	-2.685405	1.821736	-0.428661
C	-0.234967	-3.236497	-0.428661
C	1.469745	0.722848	1.859265
C	-1.360877	0.911413	1.859265
C	-0.108868	-1.634261	1.859265
C	1.469745	-0.722848	-1.859265
C	-0.108868	1.634261	-1.859265
C	-1.360877	-0.911413	-1.859265

D_{3S} SR

Os	1.656511	0.000000	0.000000
Os	-0.828255	1.434581	0.000000
Os	-0.828255	-1.434581	0.000000
O	3.586378	-2.280508	0.672202
O	0.181788	4.246149	0.672202
O	-3.768167	-1.965641	0.672202
O	3.586378	2.280508	-0.672202
O	-3.768167	1.965641	-0.672202
O	0.181788	-4.246149	-0.672202
O	1.608461	0.909453	2.961923
O	-1.591840	0.938242	2.961923
O	-0.016621	-1.847695	2.961923
O	1.608461	-0.909453	-2.961923
O	-0.016621	1.847695	-2.961923
O	-1.591840	-0.938242	-2.961923
C	2.863136	-1.435286	0.394341
C	-0.188574	3.197191	0.394341
C	-2.674562	-1.761906	0.394341
C	2.863136	1.435286	-0.394341
C	-2.674562	1.761906	-0.394341
C	-0.188574	-3.197191	-0.394341
C	1.568655	0.564249	1.871147

C	-1.272981	1.076370	1.871147
C	-0.295673	-1.640619	1.871147
C	1.568655	-0.564249	-1.871147
C	-0.295673	1.640619	-1.871147
C	-1.272981	-1.076370	-1.871147

D_{3S} SO

Os	1.656208	0.000000	0.000000
Os	-0.828104	1.434318	0.000000
Os	-0.828104	-1.434318	0.000000
O	3.586332	-2.278391	0.671421
O	0.179978	4.245051	0.671421
O	-3.766311	-1.966659	0.671421
O	3.586332	2.278391	-0.671421
O	-3.766311	1.966659	-0.671421
O	0.179978	-4.245051	-0.671421
O	1.609504	0.909033	2.960993
O	-1.591998	0.939354	2.960993
O	-0.017506	-1.848388	2.960993
O	1.609504	-0.909033	-2.960993
O	-0.017506	1.848388	-2.960993
O	-1.591998	-0.939354	-2.960993
C	2.862431	-1.433353	0.394099
C	-0.189895	3.195615	0.394099
C	-2.672536	-1.762262	0.394099
C	2.862431	1.433353	-0.394099
C	-2.672536	1.762262	-0.394099
C	-0.189895	-3.195615	-0.394099
C	1.569652	0.563535	1.870245
C	-1.272862	1.077591	1.870245
C	-0.296790	-1.641126	1.870245
C	1.569652	-0.563535	-1.870245
C	-0.296790	1.641126	-1.870245
C	-1.272862	-1.077591	-1.870245

D_{3h} NR

Os	0.808680	1.400675	0.000000
Os	0.808680	-1.400675	0.000000
Os	-1.617360	0.000000	0.000000
O	-0.275890	4.314200	0.000000
O	3.874152	-1.918172	0.000000
O	-3.598262	-2.396028	0.000000
O	3.874152	1.918172	0.000000
O	-0.275890	-4.314200	0.000000
O	-3.598262	2.396028	0.000000
O	0.874788	1.515177	3.123108
O	0.874788	-1.515177	3.123108
O	-1.749575	0.000000	3.123108
O	0.874788	1.515177	-3.123108
O	0.874788	-1.515177	-3.123108
O	-1.749575	0.000000	-3.123108

C	0.108717	3.237790	0.000000
C	2.749649	-1.713047	0.000000
C	-2.858367	-1.524743	0.000000
C	2.749649	1.713047	0.000000
C	0.108717	-3.237790	0.000000
C	-2.858367	1.524743	0.000000
C	0.794535	1.376175	1.991615
C	0.794535	-1.376175	1.991615
C	-1.589070	0.000000	1.991615
C	0.794535	1.376175	-1.991615
C	0.794535	-1.376175	-1.991615
C	-1.589070	0.000000	-1.991615

D_{3h} SR

Os	0.832382	1.441729	0.000000
Os	0.832382	-1.441729	0.000000
Os	-1.664765	0.000000	0.000000
O	-0.299415	4.286981	0.000000
O	3.862342	-1.884189	0.000000
O	-3.562927	-2.402792	0.000000
O	3.862342	1.884189	0.000000
O	-0.299415	-4.286981	0.000000
O	-3.562927	2.402792	0.000000
O	0.877980	1.520705	3.093099
O	0.877980	-1.520705	3.093099
O	-1.755959	0.000000	3.093099
O	0.877980	1.520705	-3.093099
O	0.877980	-1.520705	-3.093099
O	-1.755959	0.000000	-3.093099
C	0.121606	3.220372	0.000000
C	2.728122	-1.715500	0.000000
C	-2.849727	-1.504873	0.000000
C	2.728122	1.715500	0.000000
C	0.121606	-3.220372	0.000000
C	-2.849727	1.504873	0.000000
C	0.825468	1.429752	1.954207
C	0.825468	-1.429752	1.954207
C	-1.650936	0.000000	1.954207
C	0.825468	1.429752	-1.954207
C	0.825468	-1.429752	-1.954207
C	-1.650936	0.000000	-1.954207

D_{3h} SO

Os	0.832465	1.441871	0.000000
Os	0.832465	-1.441871	0.000000
Os	-1.664929	0.000000	0.000000
O	-0.296965	4.286374	0.000000
O	3.860592	-1.886007	0.000000
O	-3.563626	-2.400367	0.000000
O	3.860592	1.886007	0.000000
O	-0.296965	-4.286374	0.000000
O	-3.563626	2.400367	0.000000

O	0.878370	1.521381	3.092025
O	0.878370	-1.521381	3.092025
O	-1.756740	0.000000	3.092025
O	0.878370	1.521381	-3.092025
O	0.878370	-1.521381	-3.092025
O	-1.756740	0.000000	-3.092025
C	0.123494	3.219375	0.000000
C	2.726314	-1.716636	0.000000
C	-2.849807	-1.502739	0.000000
C	2.726314	1.716636	0.000000
C	0.123494	-3.219375	0.000000
C	-2.849807	1.502739	0.000000
C	0.825994	1.430664	1.953042
C	0.825994	-1.430664	1.953042
C	-1.651989	0.000000	1.953042
C	0.825994	1.430664	-1.953042
C	0.825994	-1.430664	-1.953042
C	-1.651989	0.000000	-1.953042

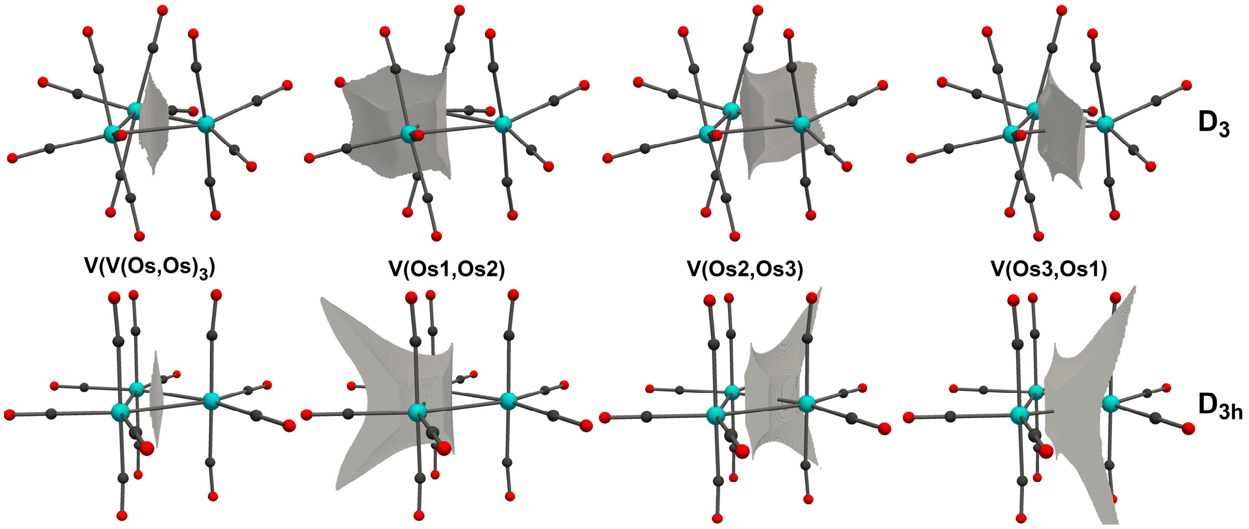

Figure S1. ELF basins for Os_3CO_{12} clusters with D_3 (top) and D_{3h} (bottom) symmetries.

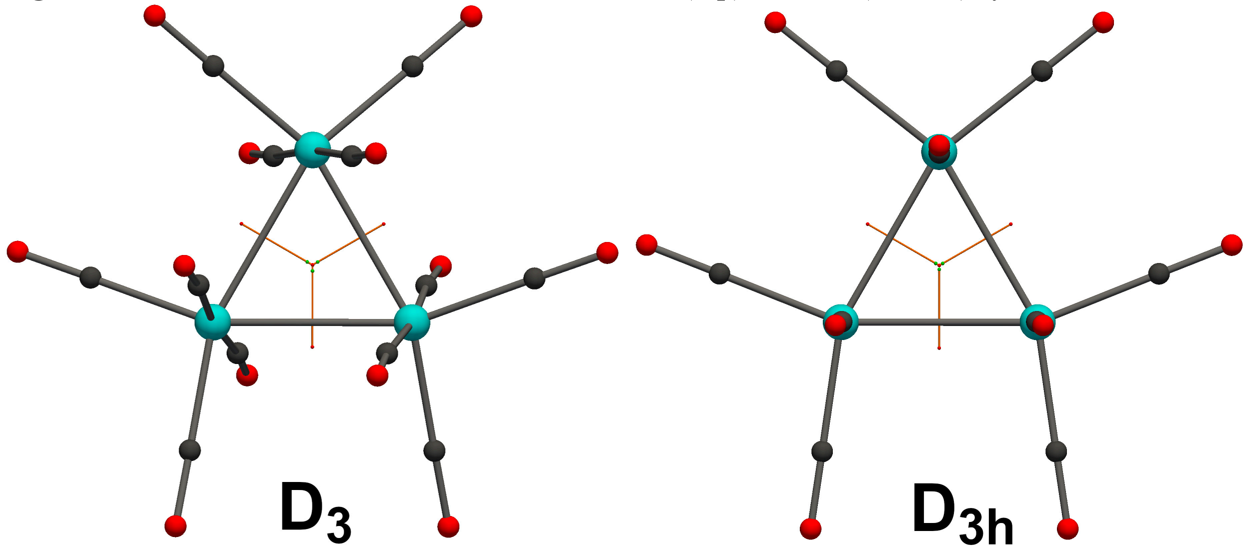

Figure S2. Critical points between V(Os,Os) and V(V(Os,Os)$_3$) basins.
